# Supplementary material for: ECG pathology and its association with death in critically ill COVID-19 patients, a cohort study
Source: PLoS One. 2021 Dec 14;16(12):e0261315. doi: 10.1371/journal.pone.0261315 (PMC8670711; doi:10.1371/journal.pone.0261315)
Supplement: S1 Appendix — (DOCX) [file pone.0261315.s001.docx]

**S1 Appendix. Sensitivity analysis.**

To investigate the impact of the predefined time restriction for when ECGs could be recorded in relation to intensive care unit admission on the main results, a sensitivity analysis was performed. In this analysis, ECGs recorded within one day from ICU admission were included (n=60). At 30-days follow-up, 17 patients (28%) had died in this sub-cohort. Among patients with normal ECG (n=38), 30-day mortality was 18%. Among patients with an ECG consistent with prior myocardial infarction (MI) pattern (n=9), mortality was 67% and among patients with ST-T pathology (n=13), mortality was 38%. Logistic regression analysis was performed for composite ECG pathology (S1 Table). Multivariable analyses were adjusted for Simplified Acute Physiology Score 3. The estimates of the logistic regression analyses were similar to the analyses of the entire study cohort, although univariable analysis of ST-T pathology was not statistically significant (P=0.15).

**S1 Table. Analysis of odds ratio for death** **within 30 days of intensive care unit admission.**

|  |  |  | **Univariable analysis** | | **Multivariable analysis** | |
| --- | --- | --- | --- | --- | --- | --- |
| **ECG-abnormality** | **Survived (n=43)** | **Died (n=17)** | **OR (95% CI)** | **P-value** | **OR (95% CI)** | **P-value** |
| **Composite abnormalities** |  |  |  |  |  |  |
| Normal ECG | 31 (72%) | 7 (41%) | Ref. | n.a. | Ref. | n.a. |
| Prior MI pattern | 3 (7%) | 6 (35%) | 9.14 (1.95-52.7) | 0.007 | 7.94 (1.51-51.7) | 0.019 |
| ST-T pathology | 8 (19%) | 5 (41%) | 2.78 (0.67-11.2) | 0.15 | 1.49 (0.14-13.1) | 0.72 |

Data are presented as absolute numbers (percentages). Logistic regression with normal ECG as reference (n=38) was performed for composite ECG variables and restricted to patients with ECG recorded within one day of intensive care unit admission (n=60). Multivariable analysis adjusted for Simplified Acute Physiology Score 3. CI: Confidence interval. Prior MI (myocardial infarction) pattern includes ECG with Q-wave and/or poor R-wave progression. ST-T-pathology includes ECG with ST-elevation, ST-depression or T-wave inversion.
